# Molecular Characterization of *Clostridium botulinum* Harboring the *bont*/B7 Gene

**DOI:** 10.1089/fpd.2018.2600

**Published:** 2019-06-03

**Authors:** Jessica L. Halpin, Janet K. Dykes, Lee Katz, Dominick A. Centurioni, Michael J. Perry, Christina T. Egan, Carolina Lúquez

**Affiliations:** ^1^ Centers for Disease Control and Prevention, Enteric Diseases Laboratory Branch, National Botulism and Enteric Toxins Team, Atlanta, Georgia.; ^2^ Center for Food Safety, College of Agricultural and Environmental Sciences, University of Georgia, Griffin, Georgia.; ^3^ New York State Department of Health, Wadsworth Center, David Axelrod Institute, Albany, New York.

**Keywords:** botulism, proteolytic, C. botulinum, subtype B7, bont/B7, infant botulism

## Abstract

*Clostridium botulinum* produces botulinum neurotoxin (BoNT), which is the causative agent of botulism, a rare but serious disease that can result in death if not treated. Infant botulism occurs when *C. botulinum* colonizes the intestinal tract of infants and produces BoNT. It has been proposed that infants under the age of 1 year are uniquely susceptible to colonization by *C. botulinum* as their intestinal microbiota is not fully developed and provides little competition, allowing *C. botulinum* to thrive and produce BoNT in the gut. There are seven well-characterized serotypes (A–G) of BoNT identified by the ability of specific antitoxins to neutralize BoNTs. Molecular technology has allowed researchers to narrow these further into subtypes based on nucleic acid sequences of the botulinum toxin (*bont*) gene. One of the most recently recognized subtypes for *bont*/B is subtype *bont*/B7. We identified through whole genome sequencing five *C. botulinum* isolates harboring *bont*/B7 from CDC's strain collection, including patient isolates and an epidemiologically linked isolate from an opened infant formula container. In this study, we report the results of whole genome sequencing analysis of these *C. botulinum* subtype bont/B7 isolates. Average nucleotide identity and high quality single nucleotide polymorphism (hqSNP) analysis resulted in two major clades. The epidemiologically linked isolates differed from each other by 2–6 hqSNPs, and this clade separated from the other isolates by 95–119 hqSNPs, corroborating available epidemiological evidence.

## Introduction

*C**lostridium botulinum* is a Gram-positive spore-forming obligate anaerobe that is ubiquitous in soil (Hatheway, 1990). *C. botulinum* produces botulinum neurotoxin (BoNT), a toxin that causes botulism, a rare but serious neuroparalytic disorder that can result in death if untreated. Four naturally occurring forms of botulism have been described: foodborne botulism, infant botulism, wound botulism, and adult intestinal colonization (Sobel, 2005). Foodborne botulism is caused by ingestion of foods contaminated with BoNT. Wound botulism occurs when an open wound is contaminated with *C. botulinum* spores, which germinate in the anaerobic environment of the wound and produce BoNT. Adult intestinal colonization is caused by colonization of the intestines by *C. botulinum* spores, which germinate and produce BoNT in the intestinal tract of persons older than 1 year. Infant botulism, also known as “floppy baby syndrome,” occurs when spores of BoNT-producing clostridia colonize the gut of infants under 1 year of age. Clinicians treat infants with BabyBIG^®^, containing human-derived anti-BoNT antibodies, and frequently the recovery is uneventful, if protracted. It has been proposed that these colonization cases originate with ingestion of spores from the environment, including dust or soil exposure within the home (Chin *et al.*, 1979; Takahashi *et al.*, 1988; Nevas *et al.*, 2005; Derman *et al.*, 2014).

To date, seven BoNT serotypes (A–G) have been defined, according to the ability to neutralize the toxin *in vivo* with polyclonal antibodies. Additional serotypes have been proposed in the literature, but consensus has not been achieved regarding them in the scientific community (Barash and Arnon, 2014; Maslanka *et al.*, 2016; Doxey *et al.*, 2018). In addition, several bivalent strains have been reported that produce two toxin types, such as A and B and B and F, as well as chimeric BoNT that exhibits properties from two different serotypes, such as C and D (Kalb *et al.*, 2015; Maslanka *et al.*, 2016). Nucleotide sequences of the botulinum toxin genes (*bont*) also show diversity within serotypes. These *bont* gene variants, denominated toxin subtypes, are identified as distinct subtypes if they encode a BoNT amino acid sequence that differs from the reference sequence by at least 2.6% (Peck *et al.*, 2017). To date, eight subtypes have been identified within serotype B, denoted as *bont*/B1-B8 (Peck *et al.*, 2017). The gene encoding components of the BoNT complexes can also differ in sequence and organization; *bont/*B genes are reported to be associated with *ntnh*, *ha70*, *ha17*, *ha33*, and *botR* genes (Rossetto *et al.*, 2014).

Kalb *et al.* (Kalb *et al.*, 2012) identified *bont*/B7 by mass spectrometric analysis of the toxin produced by strain Bac-04-07755 (accession no. JQ354985), isolated from a stool sample from an infant botulism case in New York. Strain NCTC 3807, isolated from soil from the Blue Ridge Mountains of Virginia, was also identified as harboring the *bont*/B7 gene (accession no. JN120760) (Kalb *et al.*, 2012). To date, these are the only two strains reported to produce BoNT/B7. Through whole genome sequencing, we identified five other *C. botulinum* isolates in CDC's strain collection that contained the *bont*/B7 gene. Among those isolates harboring *bont*/B7 gene, we identified an isolate from an opened container of ready-to-eat infant formula and two isolates from stool samples from an infant botulism patient, who had consumed the formula. Clinical and food isolates had nearly indistinguishable pulsotype (CDC, unpublished data) indicating a close relationship between them. To date, this is one of few instances of recovery of *C. botulinum* organism from infant formula (McHugh *et al.*, 2017) and the only instance of recovery from liquid ready-to-eat formula. In this study, we analyzed whole genome sequencing data to further characterize these *C. botulinum* isolates harboring *bont*/B7.

## Materials and Methods

### Strains

Strains used in this study are described in Table 1. Strains were grown at 35°C in Chopped Meat Glucose Starch (CMGS) broth overnight anaerobically. Growth from the CMGS broth was streaked to Egg Yolk Agar for isolation to confirm purity. Single colonies were inoculated into Trypticase Peptone Glucose Yeast broth and incubated overnight anaerobically at 35°C. The presence of *C. botulinum* genes and toxin in the isolate from New York (BAC04- 07755) were detected by multiplex polymerase chain reaction and mass spectrometry as described previously (Davis *et al.*, 2016; Perry *et al.*, 2017).

**Table 1.** Clostridium Botulinum Subtype B7 Strains Used in This Study

*Strain ID*	*Location*	*Source*	*Year*
CDC37496	District of Columbia	Stool	1982
CDC37498	District of Columbia	Infant formula (opened container)	1982
CDC37513	District of Columbia	Stool	1982
BAC-04-07755	New York	Stool	2004
CDC68158	Virginia	Stool	2013
CDC69068	New Jersey	Stool	2014

All strains were associated with infant botulism cases.

### Genomic DNA extraction

Genomic DNA was extracted from 8 to 9 mL of 16–24 h anaerobic growth using Epicentre (Illumina; Madison, WI) MasterPure Complete DNA and RNA Purification Kit, with a modified method. Briefly, cells were pelleted at 4°C for 10 min at 4000 rpm, supernatant discarded and pellets resuspended in Lysozyme Stock Solution (25 mM Tris-HCl, pH 8.0, 2.5 mM 0.5 M EDTA, 10 mL Triton X-100, 20 mg/mL Lysozyme from chicken egg white), and incubated for 15 min in a preheated 37°C water bath. A measure of 300 μL of undiluted 2X T& C buffer and 3 μL of RNase A (Qiagen; Redwood City, CA) were added to the cell suspension and incubated for 10 min in a preheated 57°C water bath, followed by addition of 3 μL of Proteinase K (Invitrogen/Thermo Fisher; Waltham, MA) and another 10 min incubation in the 57°C water bath. To extract DNA, 350 μL of MPC Protein Precipitation Buffer was added to the cell solution and then centrifuged at 4°C for 10 min at 4000 rpm. Supernatants were collected and each added to 1 mL of 99% isopropanol. Solutions were gently mixed and DNA precipitates were collected, washed with 1 mL of 70% ethanol, and rehydrated overnight in 10 mM Tris. The DNA extracts were then filtered through 0.1 μM centrifugal filters (MilliporeSigma; Burlington, MA).

### Whole genome shotgun sequencing

Libraries were constructed with the Kapa Biosciences 200 Base Pair Kit (Roche; Wilmington, MA), pooled and diluted to obtain an equimolar total concentration of 100 pM, and then were templated and enriched using the Ion Torrent Chef (Thermo Fisher; Waltham, MA) automated system and Ion Hi-Q (Thermo Fisher; Waltham, MA) chemistry. Sequencing was completed for CDC68158, CDC69068, CDC37496, CDC37498, and CDC37513 using the Ion Torrent Personal Genome machine (Thermo Fisher; Waltham, MA) and resulted in an average coverage of 49.5. × (24 × –94 × ). The New York Department of Health isolated BAC-04-07755, performed DNA extractions, created Illumina sequencing libraries using the Nextera XT Kit (Illumina; San Diego, CA), and sequenced the isolated on the MiSeq (Illumina) instrument using 2 × 250 chemistry.

### Bioinformatics

Read quality was assessed using FastQCv.11.3 (Leggett *et al.*, 2013), and reads were assembled using SPAdes v.3.1.0 (Bankevich *et al.*, 2012). Assemblies were assessed using Quast v. 4.3 (Gurevich *et al.*, 2013). The *bont* gene subtype was determined using the “map reads to reference” feature in CLC Genomics Workbench v.9 (Qiagen; Redwood City, CA). The read coverage across the reference may be seen in the Supplementary Figure S1. We determined the nearest available reference sequence using Average Nucleotide Identity (ANI) (Goris *et al.*, 2007). We used Mashtree to create a neighbor-joining tree with the sequences and type B reference strains. Then, we used Lyve-SET (Katz *et al.*, 2017) version 1.1.4f (settings were for single ended reads, with minimum coverage of 10, the minimum alt fraction was set to 0.75 and the allowed flanking set to 5, phages and cliffs in the pileup were masked); CG-pipeline (Kislyuk *et al.*, 2010) was used to clean the reads, and smalt was used to map them to identify high quality single nucleotide polymorphisms (hqSNPs) common to all 6 sequences and to approximate a phylogeny with bootstrap support. The sequence type (ST) of the 6 *C. botulinum* isolates harboring bont/B7 was determined by querying the Center for Genomic Epidemiology's (Denmark Technical University; Lyngby, Denmark) multilocus sequence typing (MLST) tool.

## Results

Resulting reads and assembly and mapping statistics can be found in Table 2.

**Table 2.** Sequencing Statistics for Clostridium Botulinum Subtype B7 Strains Used in This Study

*Strain ID*	*Total length*	*GC%*	*No. of reads*	*Average read length (bp)*	*De novo coverage*	*No. of reads mapped to chromosome Okra B1*	*No. of reads mapped to plasmid Okra B1*	*No. of unmapped reads*
CDC37496	4,024,675	28.1	1,933,230	194	93.8	1,830,273	83,943	19,014
CDC37498	3,905,902	28.1	1,375,102	208	71.5	1,370,930	1655	2517
CDC37513	3,998,101	28.0	639,392	201	32.1	610,995	23,553	4844
BAC-04-07755	4,015,174	28.1	R1 1,474,900	250	184.4	2,843,494	81,031	25,275
			R2 1,474,900					
CDC68158	4,050,546	28.2	505,860	188	23.8	479,275	22,901	3684
CDC69068	4,019,721	28.1	491,333	217	26.7	37,995	651	452,687

Seven-gene MLST is a profiling methodology that can be used to characterize and group strains of *C. botulinum* into ST profiles (Jacobson *et al.*, 2008a). All six isolates harboring *bont*/B7 were identified as ST-34.

We also analyzed the genes encoding components of the BoNT complexes: *ntnh*, *ha70*, *ha17*, *ha33*, and *botR* genes. Sequences from two isolates had sufficient coverage to reconstruct the sequences from these accessory genes. We used the CLC genomics map reads to reference feature to extract the consensus sequence for the genes from the short reads and the NCBI BLAST (NCBI; Bethesda, MD) feature to find other similar sequences. All three *ha* genes, *botR* gene, and *ntnh* gene were 100% similar to the corresponding genes in reference strain Okra, subtype *bont*/B1. The *bont* gene clusters were found on plasmids similar to pCLD rather than the chromosome.

We used pairwise ANI to determine homology between the draft assemblies, as well as to *C. botulinum* references CDC67086, ATCC3502, and Okra. ANI values among sequences of *C. botulinum* isolates containing *bont*/B7 gene were >99% (99.24–99.90%). ANI values between genome assemblies containing *bont*/B7 gene and the serotype B reference sequence Okra, which harbors a *bont*/B1 gene, were >99% (99.23–99.98%), while ANI values for CDC67086 and ATCC3502 were <98%.

**FIG. 1.**
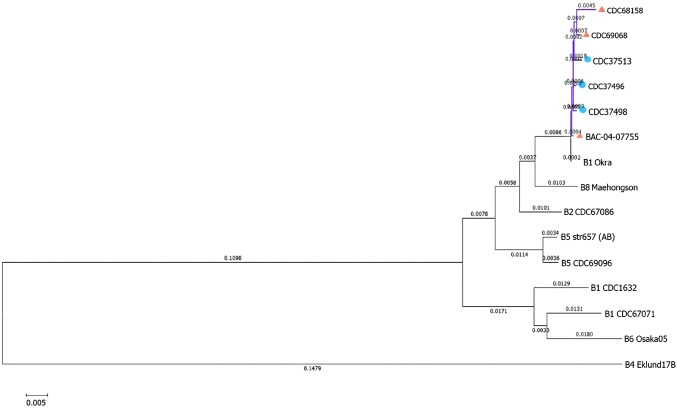
Neighbor-joining tree drawn from Mash distances containing well-characterized *Clostridium botulinum* reference strains and the six subtype B7 isolates used in this study. Circles represent the formula isolate and clinical isolates. Triangles represent the type strain and unrelated infant cases that produced an isolate harboring *bont*/B7.

A neighbor-joining tree generated with MASH distances (Ondov *et al.*, 2016), which included *C. botulinum* type B reference sequences revealed a single clade containing the six *C. botulinum* subtype B7 sequences and the reference strain Okra (subtype B1) (Fig. 1). This tree also shows the close relationship between the two *C. botulinum* subtype B7 isolated from stool specimens (CDC37496 and CDC37513) and the associated isolate CDC37498 obtained from infant formula.

**FIG. 2.**
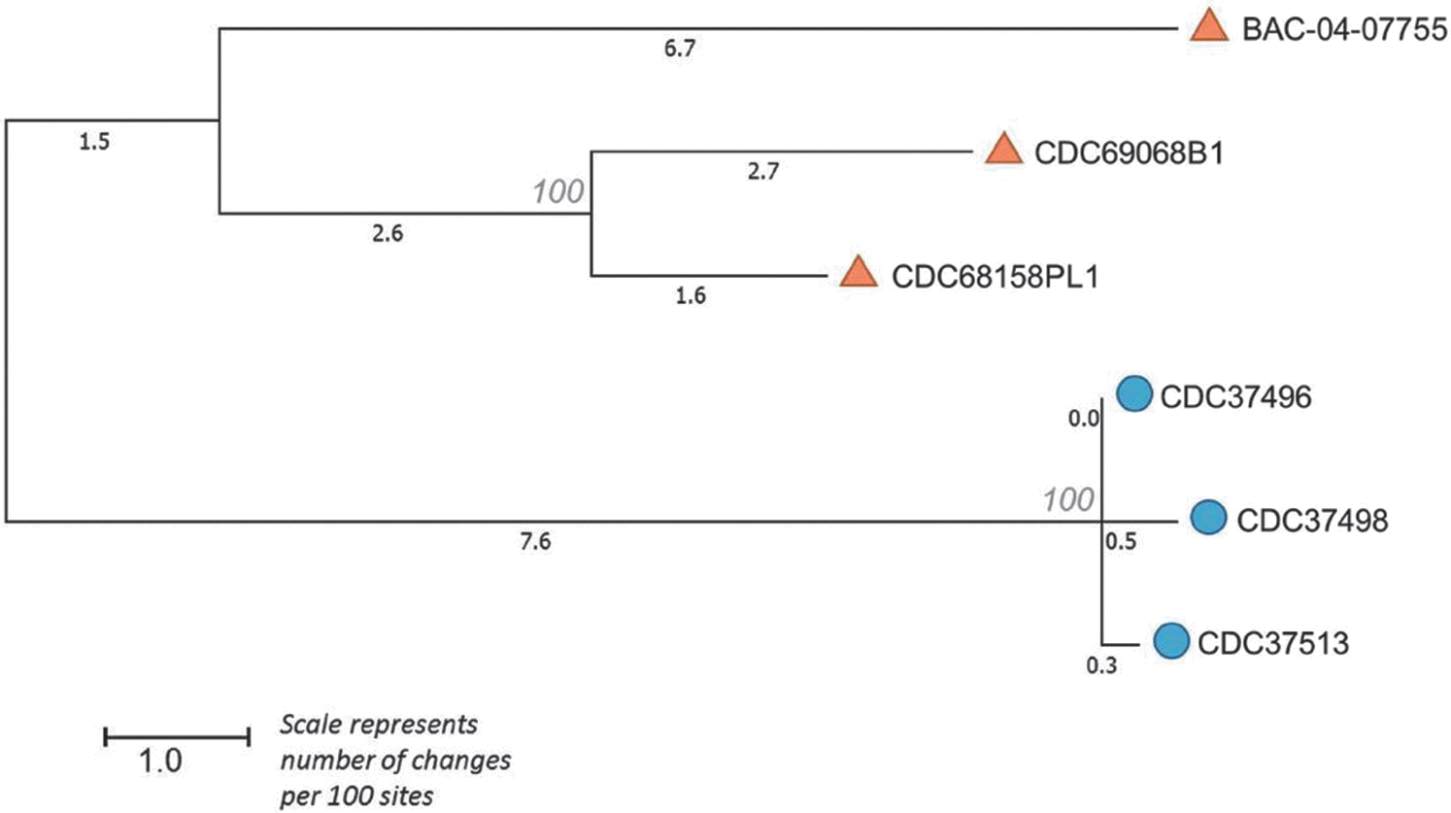
High quality SNP tree generated by Lyve-SET with bootstrap support. Circles represent the formula isolate and clinical isolates. Triangles represent the type strain and unrelated infant cases that produced an isolate harboring *bont*/B7. SNP, single nucleotide polymorphism.

To further characterize these isolates, we used Lyve-SET to identify hqSNPs. Lyve-SET identified 180 hqSNPs that were used to approximate a phylogenetic tree with bootstrap support (Fig. 2). The resulting tree contained two clades, one of which contained CDC37496, CDC37498, and CDC37513. There were 2–6 hqSNP differences between the three isolates. By contrast, there were between 95 and 119 hqSNP differences between the two clades and 32–87 hqSNP differences within the second clade.

Draft assemblies and reads were uploaded to GenBank and the sequence read archive under the following accessions: SRR6451451, SRR6451452, SRR6451450, SRR6462918, SRR6462919, SRR8444826, POTI00000000, POTJ00000000, POTK00000000, POTG00000000, POTH00000000, SCKF00000000.

## Discussion

Since Kalb *et al.* reported subtype *bont*/B7 in 2012 (Kalb *et al.*, 2012), there have been no new descriptions in the literature of strains harboring this toxin gene subtype. We undertook this study to assess the diversity of *C. botulinum* strains harboring the *bont*/B7 gene. All of the isolates described herein were obtained from stool or food specimens associated with infant botulism cases. Of note, these botulism cases occurred in the east coast of the United States, where serotype A is more prevalent (Centers for Disease Control and Prevention, 1998).

Traditional 7-gene MLST (Jacobson *et al.*, 2008b) results indicated that all six *C. botulinum* subtype B7 isolates were members of ST-34. The PubMLST database has only three *C. botulinum* type B strains in this group: strain 407 isolated in Japan in 1984, the reference strain Okra from the United States, and strain NCTC7273 from the United Kingdom isolated in 1947. Interestingly, these three strains harbor *bont*/B1 gene rather than *bont*/B7. Both of these toxin subtypes are carried on plasmids; thus, it is not surprising that these strains share the same ST. Strains Okra and 407 have a nearly identical pulsotype as well and have been shown to share a multilocus variable number tandem repeat profile (Umeda *et al.*, 2013).

We were able to reconstruct the three *ha* genes, *botR* gene, and *ntnh* gene from the short read sequence data for CDC37496 and CDC37513. These were all most closely related to *bont*/B1 accessory genes from CDC1632 (CP013243.1) with 99% identity and did not display any unusual characteristics.

The neighbor-joining tree generated from MASH distances (Ondov *et al.*, 2016) confirmed the relationships seen with other methods (Fig. 1). All six isolates described herein that harbor the *bont*/B7 gene formed a single clade with the reference strain Okra, which harbors *bont*/B1 gene. However, the underlying genetic backbone of these strains seems to be somewhat diverse, with a wide range of hqSNP differences between the two major clades (Fig. 2).

Isolates CDC37496 and CDC37513 were nearly indistinguishable by hqSNP analysis from the epidemiologically linked isolate CDC37498, recovered from an open container of liquid infant formula. *C. botulinum* has been isolated from containers of opened powder infant formula from the home of patients with infant botulism; however, dairy powders have not been satisfactorily implicated as the cause of a case of infant botulism (McHugh *et al.*, 2017). Although the three isolates showed high similarity by hqSNP analysis, indicating a single strain or common ancestor, because the container had been opened, it is not possible to determine whether the formula was contaminated during manufacture or during use in the home. Moreover, the infant could have acquired the spores from the environment independently from ingesting the formula.

## Conclusions

Whole genome sequence analysis provides a fast and thorough mechanism for exploring characteristics of newly identified subtypes. The three epidemiologically linked clinical and infant formula isolates (CDC37496, CDC37498, and CDC37513) were distinguishable from the isolates with no epidemiological link, indicating that hqSNPs revealed by the Lyve-SET algorithm can be a useful tool for analyzing closely related *C. botulinum* strains.

## Supplementary Material

Supplemental data
