# Supplementary material for: Molecular Characterization of Clostridium botulinum Harboring the bont/B7 Gene
Source: Foodborne Pathog Dis. 2019 Jun 3;16(6):428–33. doi: 10.1089/fpd.2018.2600 (PMC6585170; doi:10.1089/fpd.2018.2600)
Supplement: Supplemental data [file Supp_Fig1.pdf]

## Supplementary Data

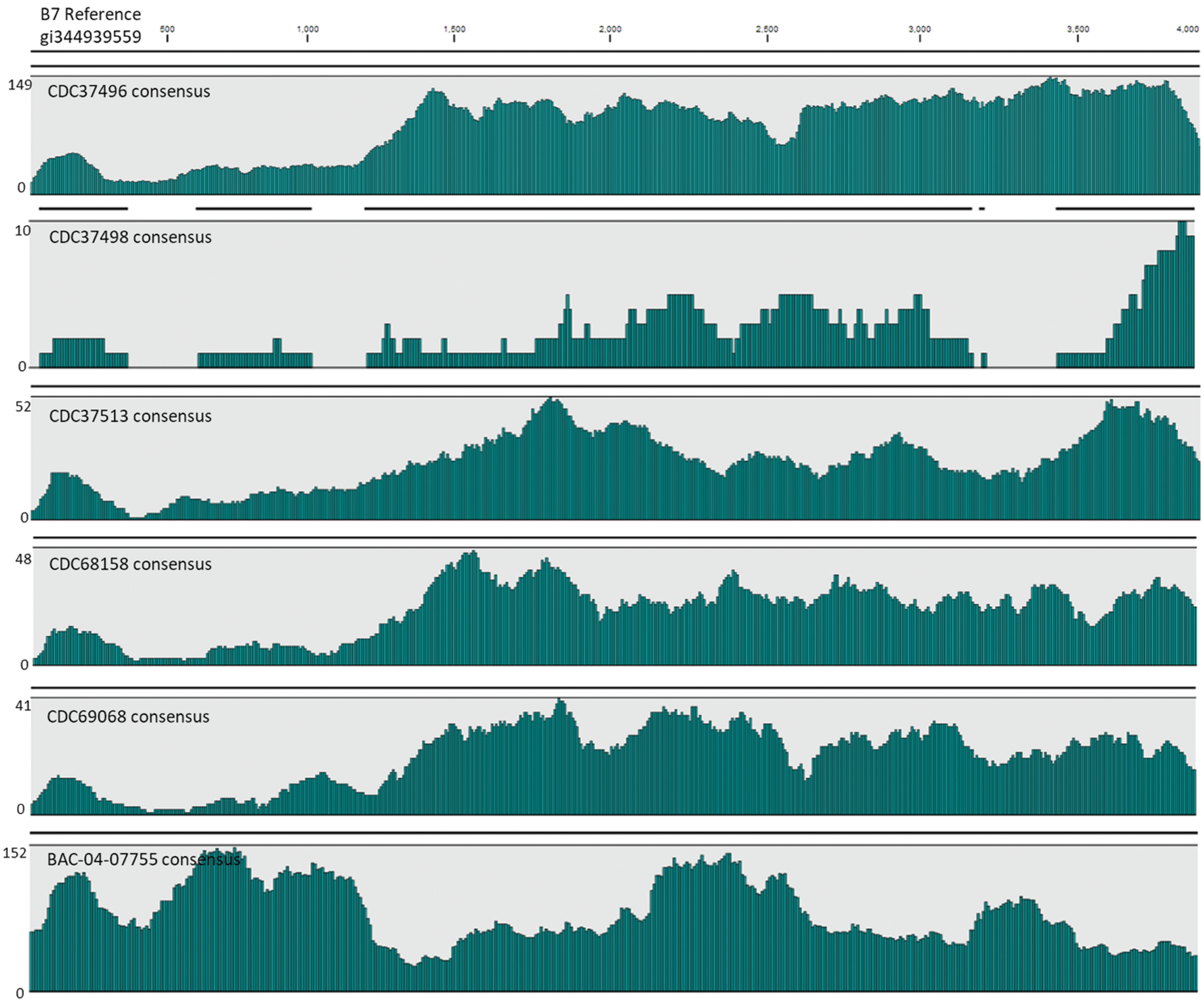


**SUPPLEMENTARY FIG. S1.** Graph of coverage for reads mapping to the reference B7 toxin gene (gi 344939559). Coverage scale is indicated at the left of each map, and the name of each read set is located at the top left of each map. The line at the top of the map is the consensus sequence, indicating any breaks compared to the reference sequence.
